# Humidity and measurement of volatile propofol using MCC-IMS (EDMON)

**DOI:** 10.1007/s10877-022-00907-0

**Published:** 2022-09-21

**Authors:** Tobias Teucke, F Maurer, LM Müller-Wirtz, T Volk, DI Sessler, S Kreuer

**Affiliations:** 1 CBR - Center of Breath Research, Department of Anaesthesiology, Intensive Care and Pain Therapy, Faculty of Medicine, Saarland University Medical Center, Saarland University, 66421 Homburg, Saar, Germany; 2 Department of OUTCOMES RESEARCH, Anaesthesiology Institute, Cleveland Clinic, Cleveland, OH USA

**Keywords:** Anaesthesia, Exhaled propofol, Ion mobility spectrometry, MCC-IMS, EDMON, Drug monitoring.

## Abstract

The bedside **E**xhaled **D**rug **MON**itor – EDMON measures exhaled propofol in ppb_v_ every minute based on multi-capillary column – ion mobility spectrometry (MCC-IMS). The MCC pre-separates gas samples, thereby reducing the influence of the high humidity in human breath. However, preliminary analyses identified substantial measurement deviations between dry and humid calibration standards. We therefore performed an analytical validation of the EDMON to evaluate the influence of humidity on measurement performance. A calibration gas generator was used to generate gaseous propofol standards measured by an EDMON device to assess linearity, precision, carry-over, resolution, and the influence of different levels of humidity at 100% and 1.7% (without additional) relative humidity (reference temperature: 37°C). EDMON measurements were roughly half the actual concentration without additional humidity and roughly halved again at 100% relative humidity. Standard concentrations and EDMON values correlated linearly at 100% relative humidity (R²=0.97). The measured values were stable over 100min with a variance ≤ 10% in over 96% of the measurements. Carry-over effects were low with 5% at 100% relative humidity after 5min of equilibration. EDMON measurement resolution at 100% relative humidity was 0.4 and 0.6 ppb_v_ for standard concentrations of 3 ppb_v_ and 41 ppb_v_. The influence of humidity on measurement performance was best described by a second-order polynomial function (R²≥0.99) with influence reaching a maximum at about 70% relative humidity. We conclude that EDMON measurements are strongly influenced by humidity and should therefore be corrected for sample humidity to obtain accurate estimates of exhaled propofol concentrations.

## Introduction

Propofol is the most used intravenous anaesthetic for induction and maintenance of general anaesthesia and sedation. Anaesthetic effect can be measured by electroencephalographic systems like the Bispectral Index monitor (BIS). However, the electroencephalogram is influenced by opioids and neuromuscular blocking agents, and is subject to internal and external influences such as eye blinking or sensor movement which add noise to the signal [1–3]. An alternative approach is to incorporate pharmacokinetic models into infusion pumps which estimate propofol plasma and effect-site concentrations during target-controlled infusions (TCI) [4]. Despite recent development of a new pharmacokinetic model that include data from thousands of patients [5], errors still exceed 22% [6–8].

Inappropriate dosing may cause haemodynamic instability, delayed recovery, or (rarely) intraoperative awareness [9–12]. Real-time monitoring of propofol plasma concentrations is therefore desirable. Monitoring of the exhaled concentration, as routinely performed for volatile anaesthetics, has not previously been available for propofol. Thus, optimal propofol dosing can be challenging for clinicians, and is usually largely guided by experience. One promising non-invasive approach is measurement of exhaled propofol via multi-capillary column – ion mobility spectrometry (MCC-IMS) [13–17].

Real-time propofol monitoring from exhaled breath is a rapidly developing field of research. Harrison et al. were the first to perform online measurements of exhaled propofol, reported in 2003 [18]. Exhaled concentrations correlate reasonably well with blood concentrations [14], [19–22], and various analytical methods have since been used [13], [14], [19], [20], [23–25].

High humidity in breath influences the underlying technology of ion mobility spectrometry [26–28]. A multi-capillary column, which is included in the EDMON, pre-separates the gas sample thereby moderating the influence of high humidity in human breath. However, preliminary analyses suggested that residual humidity substantially degraded measurement performance.

The EDMON monitor is primarily intended to be used in mechanically ventilated patients. During mechanical ventilation, humidity of breath samples varies depending on various factors including use of heat and moisture exchange filter and fresh gas flow [29]. Under normal non ventilated conditions exhaled breath has a relative humidity of ~ 80–90% with ~ 100% during end tidal sampling with a reference temperature of 31,5°C [30]. We therefore performed an analytical validation of the EDMON monitor with a specific focus on the influence of humidity on measurement performance.

## Materials and methods

### Experimental setup

Propofol stock solution of 35.9µg mL^-1^ was freshly prepared by dissolving propofol ≥ 97% (Sigma-Aldrich, Steinheim, Germany) in 1% v/v absolute ethanol (Sigma-Aldrich, Steinheim, Germany) in HPLC-grade water or n-hexane ≥ 99% (VWR International, Geldenaaksebaan, Belgium) for all measurements and stored in a 250 mL glass bottle. To enhance accuracy, the exact propofol concentration of the stock solution was calculated according to the weight of the added propofol determined with an analytical scale (MSA225P-1CE-DU; Sartorius, Goettingen, Germany).

Propofol standard concentrations were generated with a reference gas generator (HovaCal 4836-VOC; IAS, Oberursel, Germany) at a flow of 730 mL min^-1^ N_2_ (purity ≥ 99,95%) [31]. Propofol stock solution was dosed with 12.5 µL and 50 µL syringes (Hamilton, Planegg, Germany), while purified water (HPLC-grade; VWR, Darmstadt, Germany) was dosed with 250 µL syringes. Vaporization temperature was set to 100°C. Humidity of the resulting gas was calculated for a reference temperature of 37°C to represent physiological conditions. ViewCAL 1.2.1 (IAS, Oberursel, Germany) software was used to control the HovaCal. For standard concentrations between 1 and 30 ppb_v_ a 12.5 µL syringe was used, for higher concentrations a 50 µL syringe was used.

To prevent a loss of propofol and humidity during sampling through condensation and sorption effects, a heated (50°C) perfluoroalkoxy alkane (PFA) sample tube (IAS, Oberursel, Germany) was used and sample gas was constantly provided by the HovaCal. The EDMON was connected to this sample tube via a 1.8-meter long polytetrafluoroethylene (PTFE) tube (B.Braun Melsungen AG, Melsungen, Germany) and a stainless steel 1/8” t-piece (Swagelok, Frankfurt, Germany) open to atmospheric pressure to avoid overpressure. The EDMON was supplied with synthetic air (Air Liquide, Düsseldorf, Germany) with a purity ≥ 99.999% as drift and carrier gas. Values were generated within a minute cycle with a sampling time of 20s and a processing time of 40s. The sample was acquired with a flow of 150 mL/min. The aim was to simulate sampling inside the lung, choosing 37°C as the reference temperature and ~ 100% relative humidity for gas sampling [32], as body temperature does not change much during normal clinical conditions [33] resulting in the maximum concentration of humidity during ventilation, not including active humidification.

### Linearity

Propofol standard concentrations of 100, 90, 80, 70, 60, 50, 40, 30, 20, 10, 5 and 1 ppb_v_ with 100% relative humidity (RH; T*reference* = 37°C) and without additional humidity were generated with the HovaCal to determine linearity. Each standard concentration was measured for 30min in 1-minute intervals leading to 30 values. Initially and after each concentration step 15 blank measurements were carried out without propofol and without additional humidity to avoid carry-over effects. Limit of Detection (LOD) and Limit of Quantification (LOQ) were determined based on the standard deviation of the response and slope using the values gathered for linearity according to the European Medicines Agency guideline [34].

### Precision, Carry-over, and Resolution

Precision, carry-over, and resolution were assessed at 100% relative humidity and without additional humidity. Therefore, standard concentrations of 20 ppb_v_ and 40 ppb_v_ were sustained for 100min before being reducing to 0 ppb_v_. To analyse carry-over effects, a standard concentration of 10 ppb_v_ was maintained for 10min and then reduced to 0 ppb_v_. Resolution was graphically determined for low and high standard concentrations of 5 ppb_v_ in a range of 1–10 ppb_v_ and 40 ppb_v_ in a range of 30–50 ppb_v_ by measuring the distance between the 95% prediction interval and comparing it to values on the x-axis to receive the range in which the device can distinguish differences in concentration.

### Influence of different levels of relative humidity

Under a constant standard concentration of 30 ppb_v_, relative humidity was increased in 10% increments from 0 to 100% every 15min. The process was then reversed, with relative humidity decreasing from 100 to 0% in increments of 10%. To exclude humidity deriving from the aqueous propofol stock solution, measurements were additionally carried out with a hexane-diluted propofol stock solution.

### Data analysis and statistical analysis

EDMON data were recorded by the CLINEDMON software version 1.1 (B.Braun Melsungen AG, Melsungen, Germany). Statistical analysis was conducted in R (version 3.5.3, R Core Team, R Foundation for Statistical Computing, Vienna, Austria) with the following packages: ggsignif (0.6.0, Constantin Ahlmann-Eltze, Indrajeet Patil) and ggplot2 (3.3.0, Hadley Wickham, Thomas Lin Pedersen et al.).

Normality was determined by Shapiro-Wilk testing using standard residuals and visual inspection of histograms and quantile-quantile-plots. Normally distributed data are presented as means ± SDs, and non-normally distributed data are presented as medians and interquartile ranges. Comparisons were done using one-way ANOVA, followed by pairwise comparisons using the Tukey test. A two-sided p < 0.05 was considered statistically significant.

## Results

### Linearity

**Fig. 1:**
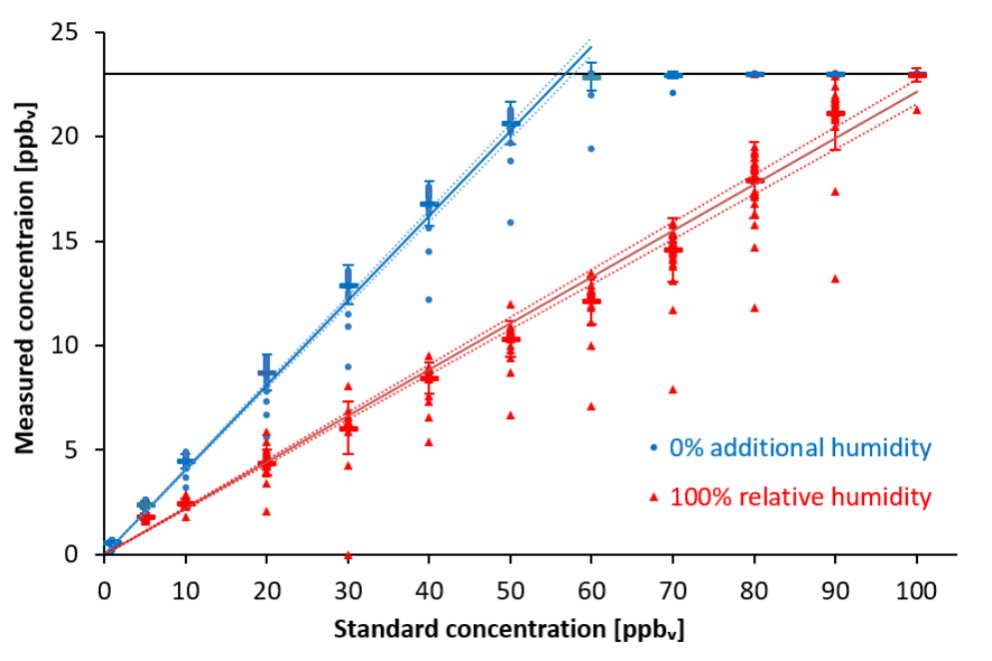
Linear calibration between HovaCal standard concentrations from 0 ppb_v_ to 100 ppb_v_ and EDMON measurements with and without additional humidity

Linearity was confirmed under 100% relative humidity for calibration standard concentrations between 5 and 100 ppb_v_ with standard deviations being ≤ 10% of the mean measured concentrations (Fig.1, red line) and only ~ 22% of the actual concentration of the calibration standard. The standard deviation exceeded 10% of the measured concentration only when the standard concentration was 90 ppb_v_. Without additional humidity, linearity was measured between 1 and 100 ppb_v_ and confirmed for calibration standard concentrations between 1 and 60 ppb_v_ with standard deviations being ≤ 10% of the mean measured concentrations (Fig.1, blue line) and only ~ 43% of the calibration standard concentration.

Mean measured concentrations were in a range of 0.57 to 23 ppb_v_ with 100% relative humidity and 1.8 to 23 ppb_v_ without additional humidity. Measured concentrations at 100% relative humidity were significantly lower than without additional humidity. Measurements of the standard concentration of 1 ppb_v_ at 100% relative humidity were excluded because measured concentrations were ≤ 0.2 ppb_v_.

Dots represent the raw data, thick dashes the means, and error bars the SDs. 95% confidence intervals are displayed as dotted lines. Measured concentrations with 100% relative humidity (red; R²= 0.97; y = 4.29x + 2.77) were significantly lower than without additional humidity (blue; R² = 0.99; y = 2.52x − 1.76) apart from standard concentrations ≤ 5 ppb_v_. The black line represents the upper measurement limit of the EDMON at 23 ppb_v_. The Limit of Detection (LOD) and Limit of Quantification (LOQ) resulted in 0.19 ppb_v_, 0.59 ppb_v_ without additional humidity and 0.79 ppb_v_, 2.4 ppb_v_ at 100% relative humidity respectively.

### Precision and carry-over

**Fig. 2:**
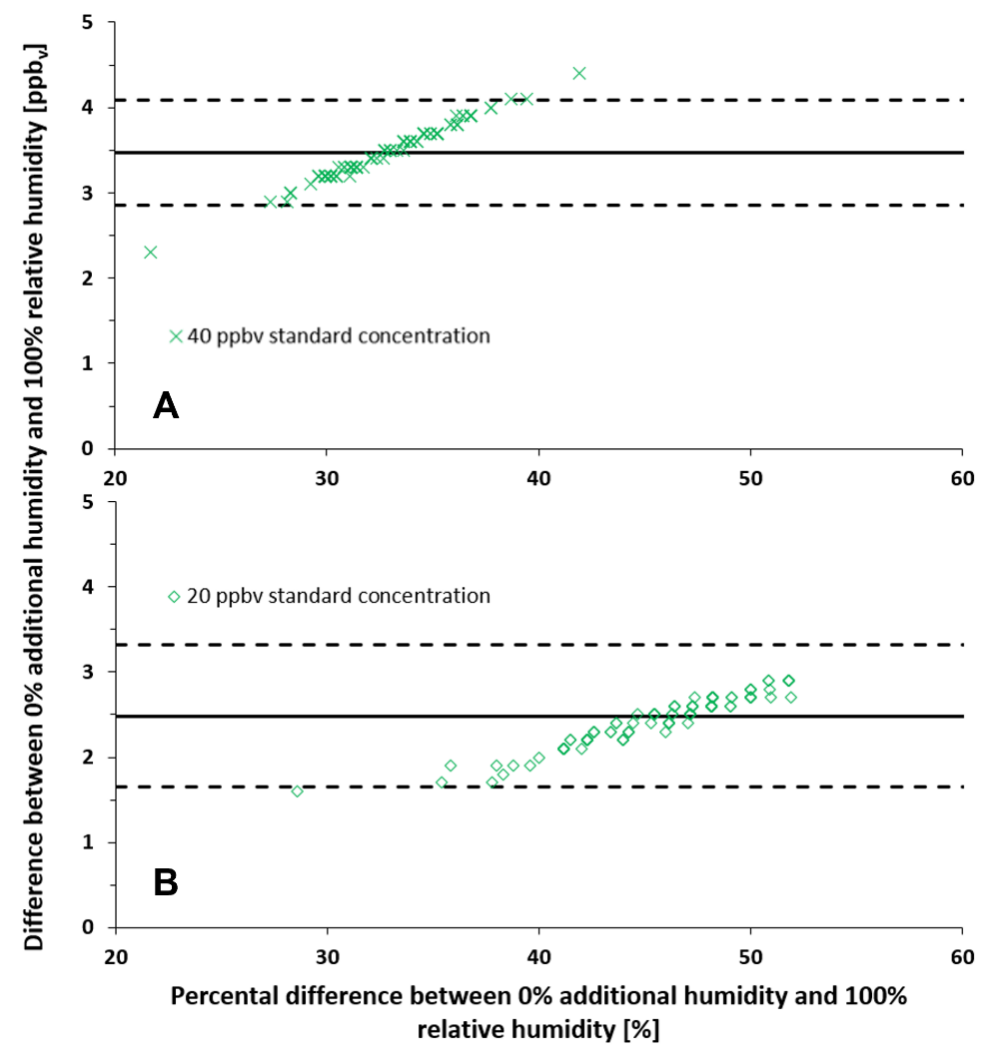
Bland-Altman plot of measured concentrations over 100min with a steady standard concentration of 20 ppb_v_ (A) and 40 ppb_v_ (B) at 100% relative humidity and without additional humidity. Upper and lower reference line are presented as dashed lines

After a 10-minute equilibration period, values were within ± 10% of the mean measured concentration for 96.5% of the measurements at 100% relative humidity and for 98.5% of the measurements without additional humidity (Fig.2A). At a standard concentration of 20 ppb_v_, measured concentrations had a relative standard deviation of 6.5% at 100% relative humidity and 5.4% without additional humidity; at 40 ppb_v_, relative standard deviations were 4.8% at 100% relative humidity and 1.2% without additional humidity (Fig.2B).

**Fig. 3:**
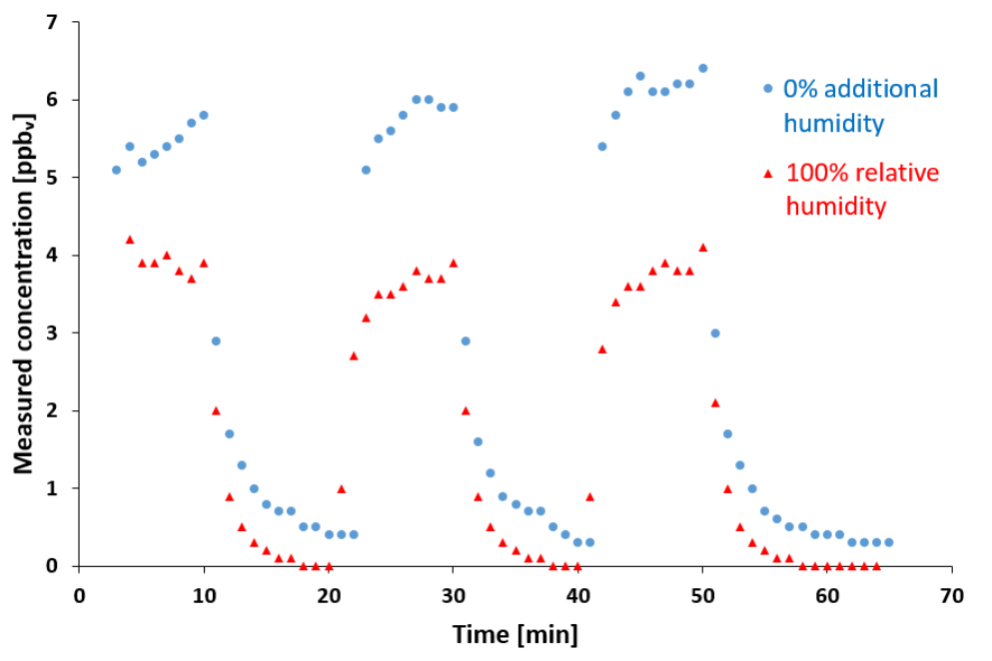
Evaluation of carry-over effects. Measured concentrations over time with 10-minute intervals of 10 ppb_v_ and 0 ppb_v_ standard concentration for measurements with 100% relative humidity and without additional humidity. Concentration changes were evaluated, after changing standard concentration from 10 ppb_v_ to 0 ppb_v_

To evaluate carry-over effects, a standard concentration of 10 ppb_v_ was generated for 10min, resulting in mean measured concentrations of 3.9, 3.6, and 3.7 ppb_v_ at 100% relative humidity, and 5.4, 5.7, and 6.1 ppb_v_ without additional humidity (Fig.3). After switching to 0 ppb_v_, mean measured concentrations dropped to 0.2, 0.2, and 0.2 ppb_v_ at 100% relative humidity and to 0.7, 0.7, and 0.6 ppb_v_ without additional humidity after 5min. Mean measured concentrations finally dropped to 0, 0, and 0 ppb_v_ at 100% relative humidity and to 0.4, 0.3, and 0.4 ppb_v_ without additional humidity after 10min. Average carry-over was 5.3% after 5min and 0% after 10min at 100% relative humidity, and 11.6% after 5min and 6.4% after 10min without additional humidity.

### Resolution

**Fig. 4:**
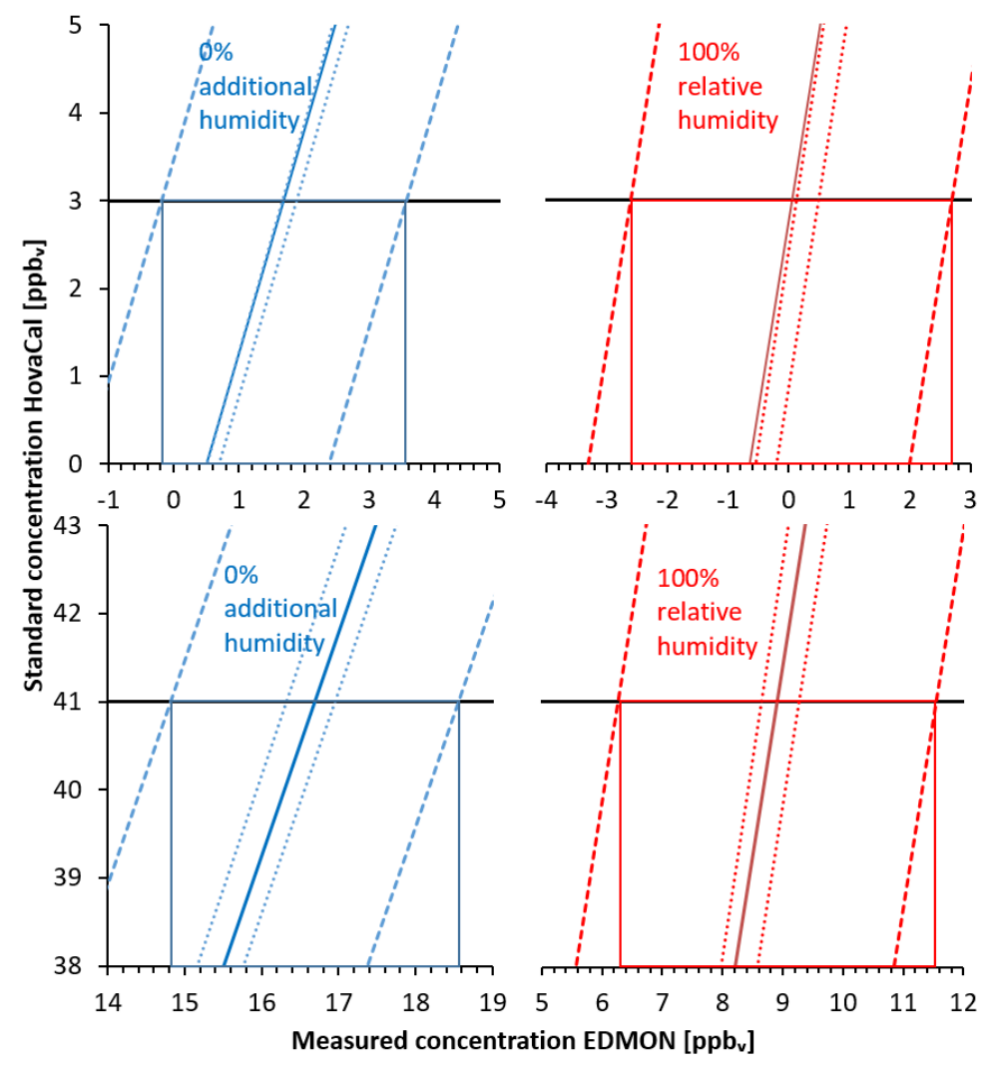
Graphical determination of resolution for standard HovaCal concentrations of 5 ppb_v_ in a range of 1–10 ppb_v_ and 40 ppb_v_ in a range of 30–40 ppb_v_.

Resolution was graphically determined based on the calibration curves given in Fig.1. Measurement resolution at 100% relative humidity was 1.3 ppb_v_ at the standard concentration 5 ppb_v_, and 3.8 ppb_v_ at 40 ppb_v_. Without additional humidity, resolution was 0.9 ppb_v_ at 5 ppb_v_, and 4 ppb_v_ at 40 ppb_v_ (Fig.4).

Solid lines represent linear regression lines. Dotted lines represent 95% confidence intervals and dashed lines represent 95% prediction intervals

### Influence of different amounts of relative humidity

**Fig. 5:**
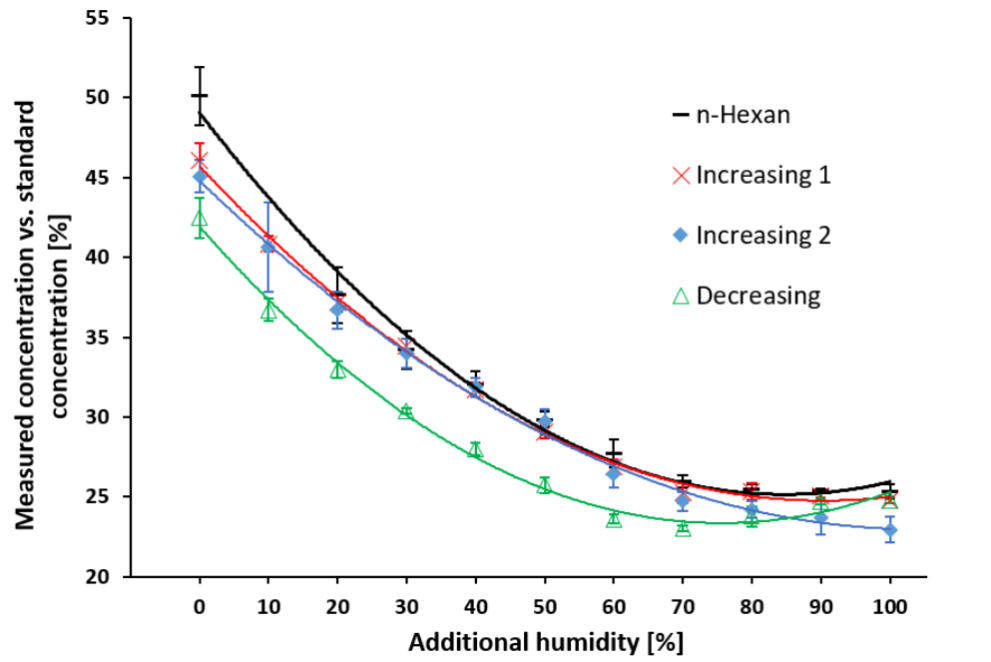
Influence of humidity on the EDMON measurement performance

Concentrations measured by the EDMON in percent of the generated standard concentrations are presented in Fig.5 as a function of the additional humidity. The measured proportion decreased following a sigmoidal function with increasing humidity. When additional humidity ranged between 70% and 100%, measured concentrations reached a plateau implicating no further influence on measurement performance at an additional humidity greater than 70%.

Two measurement series with an aqueous propofol stock solution were congruent to each other (p = 0.054), except for values at 50% additional humidity (p = 0.007). In comparison to the measurement series with a hexane-diluted stock solution, the obtained values did not significantly differ between 20% and 40% additional humidity for Increasing 1 (p = 0.64, 0.91, 0.2) and between 20% and 50% for Increasing 2 (p = 0.22, 0.99, 0.98, 0.67). Measured concentrations were significantly greater with increasing than decreasing humidity (p = 0). The mean concentrations measured by the EDMON were 24% (± 1.5) of the generated standard concentrations at 100% relative humidity and 46% (± 4) without additional humidity.

A propofol standard concentration of 30 ppb_v_ was constantly generated by a reference gas generator (HovaCal) and measured by the EDMON under increasing and decreasing relative humidity. Values are presented as the proportional difference between concentrations measured by the EDMON and the generated standard concentrations. Data are presented as means ± SDs (n = 13). Values in black were measured with propofol diluted in n-hexane instead of water. Values for 10% additional humidity were removed, as the SD was unreasonably high (9.7%; imprecision 23.5%). Increasing levels of humidity: y = 0.0026x²-0.46x + 46, R²=0.99 (red, Increasing 1); y = 0.0021x²-0.42x + 45, R²=0.99, (blue, Increasing 2); decreasing levels of humidity: y = 0.0032x²-0.49x + 42, R²=0.99 (green, Decreasing); hexane-diluted propofol standards: y = 0.0035x²-0.58x + 49, R²=0.99 (black, n-hexane).

## Discussion

Analytical validation of the EDMON device confirmed a wide linear range, high precision, good resolution, and low limits of detection and quantification which are consistent with the manufacturer’s specifications [35]. We excluded strong carry-over effects with the recommended measurement setup, confirming that the inert tubing material perfluoroalkoxy alkane and polytetrafluoroethylene is unlikely to bind propofol as previously reported [36]. Additionally, propofol is highly hydrophobic with a solubility of only 124mg/L [37], and is therefore unlikely to accumulate in condensate building up in the tubing at any humidity level.

Our results are generally consistent with a previous analytical validation of a prototype of the EDMON [38]. Linearity, precision and carry-over were comparable. However, limits of quantification (LOQ, 0.3) and detection (LOD, 0.1), and resolution (> 0.5, 2–4 ppb_v_; >0.9, 28–30 ppb_v_) were previously lower than in our validation. This is most likely caused by using raw signal intensities during the previous validation which has been disabled in recent EDMON versions. We therefore had to use the values displayed by the EDMON which are based on the manufacturer’s calibration. Access to raw signal intensities may thus have produced slightly better results for LOQ, LOD, and resolution.

Most interestingly, we found that standard concentrations, generated without additional humidity by the calibration gas generator HovaCal, differed from the concentrations measured by the EDMON device. Standard concentrations generated by the HovaCal were previously validated by liquid injection gas chromatography - mass spectrometric measurements (R²=0.89) [39]. Therefore, we presume that deviations between standard concentrations and EDMON-measured concentrations result from the manufacturer’s calibration process using permeation tubes. The calibration works by heating propofol-containing tubes to evaporate propofol at a specific rate and standard concentrations are generated by adjusting the flow rate of carrier gas around the permeation tubes [40]. The accuracy of permeation tubes is ± 5% (communication from Valco Instruments Co. Inc.) – a range that does not explain the differences we observed between standard and EDMON-measured concentrations.

We additionally observed a reduction in EDMON signal intensity of up to 50% by adding humidity to the calibration gas. EDMON signals from humid and dry gas samples thus substantially differ, additional to the already observed deviation between HovaCal-generated standard concentrations and EDMON-measured concentrations under dry conditions. Exhaled air contains up to 100% relative humidity corresponding to temperatures of exhaled air. Consequently, a calibration of the EDMON device with dry gas standards, as performed by the manufacturer, produces falsely low propofol estimates during clinical use of the EDMON. We therefore suggest using at least humidified gas standards to calibrate the EDMON to ensure accurate measurements under physiological conditions.

In contrast to Buchinger et al. [41] who state that there is no influence of humidity on the propofol signal of MCC-IMS measurements when tailing of the reactant ion peak is compensated, we observed a distinct and clinically important relationship between sample humidity and EDMON measurements which almost perfectly fit a second order polynomial function. Presumably the under-estimate results from reactant ions forming water clusters (H*(H_2_O)_n_) with high humidity [28][42]. The resulting proton affinity of 808kJ mol^-1^ ((H_2_O)_2_) is similar to the estimated proton affinity of propofol (Phenol: 817kJ mol^-1^; Toluene: 784kJ mol^-1^) [43][44] hampering ionisation. Additionally, an increase in humidity reduces the intensities of negative reactant ions and formation of negative reactant ion clusters [45].

Relative humidities between 70% and 100% roughly halved the EDMON measured concentrations compared to measurements without additional humidity. Considering the typical humidity of 27g/m³ in the mouth [46], which corresponds to 62% relative humidity (T_ref_ = 37°C), clinical samples will always contain substantial humidity, but may lay under the 70% threshold. Therefor influences on measured propofol concentrations are prone to differ during changing clinical conditions. Such, the amount of humidity in clinical samples is influenced by patient conditions, heat-and-moisture-exchanging filters, ventilation settings, active humidification, oxygen concentration, fresh mixture volume, inspiratory and expiratory settings, lung-protective ventilation, etc. [46–50]. EDMON measurements will thus be inaccurate unless corrected for humidity.

Jiang et al. [51] propose a correction for photoionization IMS that does not require measuring humidity. Specifically, the method is based on a quantitative influence of humidity on both the product ion peak (PIP) and the reactant ion peak (RIP), leading to nearly constant values for intensity ratio of Propofol/ (RIP + Propofol) at relative humidity levels of 0–98%. However, this approach may not work with the EDMON system since the in-build IMS uses atmospheric pressure chemical ionisation (APCI) [52]. Most accurately, the EDMON device should therefore monitor sample humidity to calculate propofol concentrations from calibrations at several levels of sample humidity.

In summary, linearity with and without additional humidity was observed for measured concentrations between 1 ppb_v_ and 23 ppb_v_, while imprecision over 100 measurements was ≤ 10%. Resolution is comparable for measurements with and without additional humidity whereas carry-over effects are smaller with additional humidity. The influence of humidity was substantial and results in a sigmoidal decrease in measured propofol concentration by up to 50% at relative humidities exceeding 70%. Quantitative propofol measurements must therefore be adjusted for sample humidity on-line, or at least at the beginning of the measurement, for sample humidity if it can be ensured that the humidity remains stable throughout the procedure, preferably above the 70% threshold. The equations we provide suggest an easy way to adjust for sample humidity.

## Data Availability

Not Applicable.
